# Case report: Prader–Willi syndrome and inflammatory arthritis—An important consideration

**DOI:** 10.3389/fped.2023.1102382

**Published:** 2023-03-17

**Authors:** Luca Marelli, Tomáš Dallos, Elisabetta Miserocchi, Paolo Nucci, Beatrice Tombolini, Orazio De Lucia, Maurizio Gattinara, Roberto Caporali, Achille Marino

**Affiliations:** ^1^ Eye Clinic San Giuseppe Hospital, IRCCS Multimedica Scientific Institute, Milan, Italy; ^2^ Second Department of Pediatrics, Comenius University Medical School, Bratislava, Slovakia; ^3^ School of Medicine, Vita-Salute San Raffaele University, Milan, Italy; ^4^ Division of Head and Neck, Ophthalmology Unit, IRCCS San Raffaele Scientific Institute, Milan, Italy; ^5^ Department of Biomedical, Surgical and Dental Sciences, University of Milan, Milan, Italy; ^6^ Division of Clinical Rheumatology, ASST G.Pini-CTO, Milano, Italy; ^7^ Unit of Pediatric Rheumatology, ASST G.Pini-CTO, Milan, Italy; ^8^ Department of Clinical Sciences and Community Health and Research Center for Pediatric and Adult Rheumatic Diseases (RECAP.RD), University of Milan, Milan, Italy

**Keywords:** genetic disorders, arthritis, uveitis, obesity, human growth hormone, Prader-Willi syndrome, juvenile Idiopathic arthritis

## Abstract

**Background:**

Prader–Willi syndrome (PWS) is a multisystemic genetically determined disorder. Musculoskeletal manifestations are common in most patients. We report the cases of two children with PWS who developed inflammatory arthritis, complicated with chronic anterior bilateral uveitis in one case. To our knowledge, no previous reports of such an association exist.

**Case presentation:**

Case 1 was of a 3-year-old girl diagnosed with PWS who developed arthritis of the right knee with morning stiffness, joint swelling, and limited range of motion. Other causes of arthritis were ruled out. Increased inflammatory markers, antinuclear antibody (ANA) positivity, and hypertrophic synovitis on ultrasound confirmed the diagnosis of inflammatory arthritis compatible with juvenile idiopathic arthritis (JIA). Despite the treatment with methotrexate, arthritis progressed, and etanercept was added. The patient reached and maintained articular remission while on combined MTX and etanercept treatment during 9 years of follow-up. Case 2 was of a 6-year-old boy diagnosed with PWS who developed arthritis of the right knee. Laboratory investigations showed mildly increased acute phase reactants, microcytic anemia, and ANA positivity at high titer (titer 1:1,280). Infectious and other causes of arthritis were excluded. Ultrasound confirmed the presence of joint effusion and synovial thickening, and synovial fluid analysis was consistent with inflammatory arthrosynovitis (white blood cell count of 14,200/µl) compatible with JIA. Shortly after the diagnosis, the ophthalmologic evaluation revealed the presence of bilateral anterior uveitis. Despite MTX and topical corticosteroid, ocular inflammation persisted and adalimumab was added. At the last follow-up, 9 months later, the child experienced inactivity of arthritis and uveitis with normal growth.

**Conclusions:**

We aim to raise awareness of this possible association among pediatricians since arthritis might be underestimated due to high pain tolerance, behavioral disturbances, and other musculoskeletal abnormalities in PWS patients.

## Article summary

We reported the first clinical description of the association of Prader-Willi syndrome, chronic inflammatory arthritis and anterior chronic bilateral uveitis compatible with juvenile idiopathic arthritis.

## Introduction

Prader–Willi syndrome (PWS) is a multisystemic disorder caused by the absence or lack of expression of paternally inherited genes or maternal disomy on chromosome 15 (15q11.3–q13.3) (1). Clinically, PWS is characterized by muscular hypotonia, growth deficiency, ligament laxity, hyperphagia leading to excessive body weight, hypogonadism, mental retardation, and dysmorphic features (2). The development of motor and functional skills in PWS patients may be affected by both hypotonia and obesity (3). Importantly, muscle hypotonia and impaired motor skills may further reduce mobility and potentiate morbid obesity in PWS patients. Indeed, these patients may present several musculoskeletal manifestations (4). Lower limb malalignment is one of the most reported skeletal alterations in PWS (up to 78%); unilateral and bilateral genu valgum or varum have been described. Hip dysplasia or subluxation is also frequently observed in these patients (up to 22%). About two-thirds of PWS patients develop scoliosis (with or without kyphosis) early (before 4 years of age) or during adolescence (5). Various foot abnormalities have been associated with PWS, such as pes planus, pes cavus, metatarsus adductus, and hallux valgus (4). Several factors contribute to the high prevalence of low bone density in PWS that can be prevented with endocrine therapy, vitamin D supplementation, and physical activity.

We report the cases of two children with PWS who developed inflammatory arthritis, complicated with chronic anterior bilateral uveitis in one case. To our knowledge, there have been no previous reports of such an association. Written informed consent for the publication of any potentially identifiable images or data included in this article was obtained from the parents of the herein-described patients.

## Patient presentation

### Case 1

A few weeks after birth, a Caucasian girl developed severe hypotonia, weak suction and cry reflexes, hypo/areflexia and hip and knee flexion contractures, and facial stigmata (micrognathia, almond-shaped eyes, low-set ears, short neck). The diagnosis of PWS was made based on methylation-specific polymerase chain reaction (PCR) revealing maternal disomy of the gene for the small nuclear ribonucleoprotein polypeptide N on chromosome 15. Recombinant human growth hormone (rhGH) was started at the age of 2 years, with appropriate growth and weight gain.

Ten months after the initiation of rhGH, an abdominal retroperitoneal mass was detected and surgically removed. Histologic examination disclosed a well-differentiated ganglioneuroma with no indication for chemotherapy and radiation therapy. Treatment with rhGH was temporally discontinued for 15 months during oncologic follow-up.

At the age of 3 years, the patient developed arthritis of the right knee with morning stiffness, joint swelling, and limited range of motion. A comprehensive laboratory and investigational evaluation for the differential diagnoses of arthritis was performed; infectious and neoplastic causes were ruled out. Increased inflammatory markers, antinuclear antibody (ANA) positivity, and hypertrophic synovitis on ultrasound confirmed the diagnosis of inflammatory arthritis compatible with juvenile idiopathic arthritis (JIA). Arthritis progressed with the involvement of other joints (left knee, first interphalangeal joint of the right hand, and right ankle) and was recalcitrant to systemic corticosteroids and nonsteroidal anti-inflammatory drugs.

Therefore, at the age of 4 years, subcutaneous methotrexate (MTX) was then instituted. However, arthritis persisted despite intraarticular joint injections of corticosteroids (synovial fluid was not analyzed) and MTX. Etanercept, a tumor necrosis factor inhibitor, was then added at the age of 6 years.

The patient reached and maintained articular remission while on combined MTX and etanercept treatment throughout the 9 years of follow-up (Figure 3).

During our entire observation period, there was no evidence of uveitis, and the oncologic evaluation showed no signs of ganglioneuroma progression.

### Case 2

Shortly after birth, an Egyptian boy developed hypotonia, feeding difficulties requiring enteral nutrition, and notable bilateral cryptorchidism. The presence of paternal deletion of chromosome 15 long arm on methylation-specific PCR and array comparative genomic hybridization confirmed the presumptive clinical diagnosis of PWS. The treatment with rhGH was started at the age of 1.8 years.

**Figure 1:**
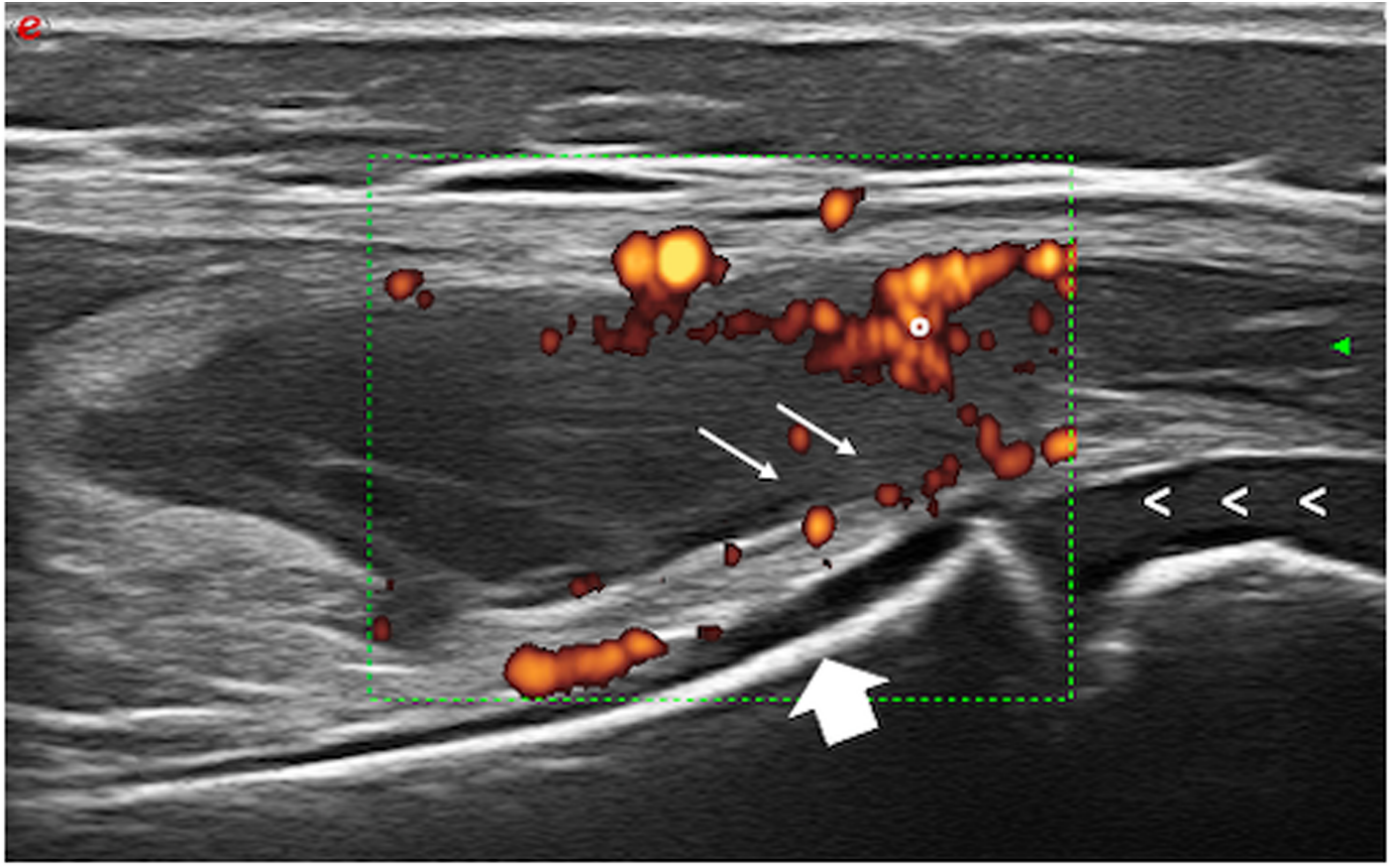
Knee ultrasound of case 2 showing active synovitis. °, Power Doppler signal of synovial tissue; arrows, synovial hypertrophy; <, growing cartilage; big arrow, bone.

**Figure 2:**
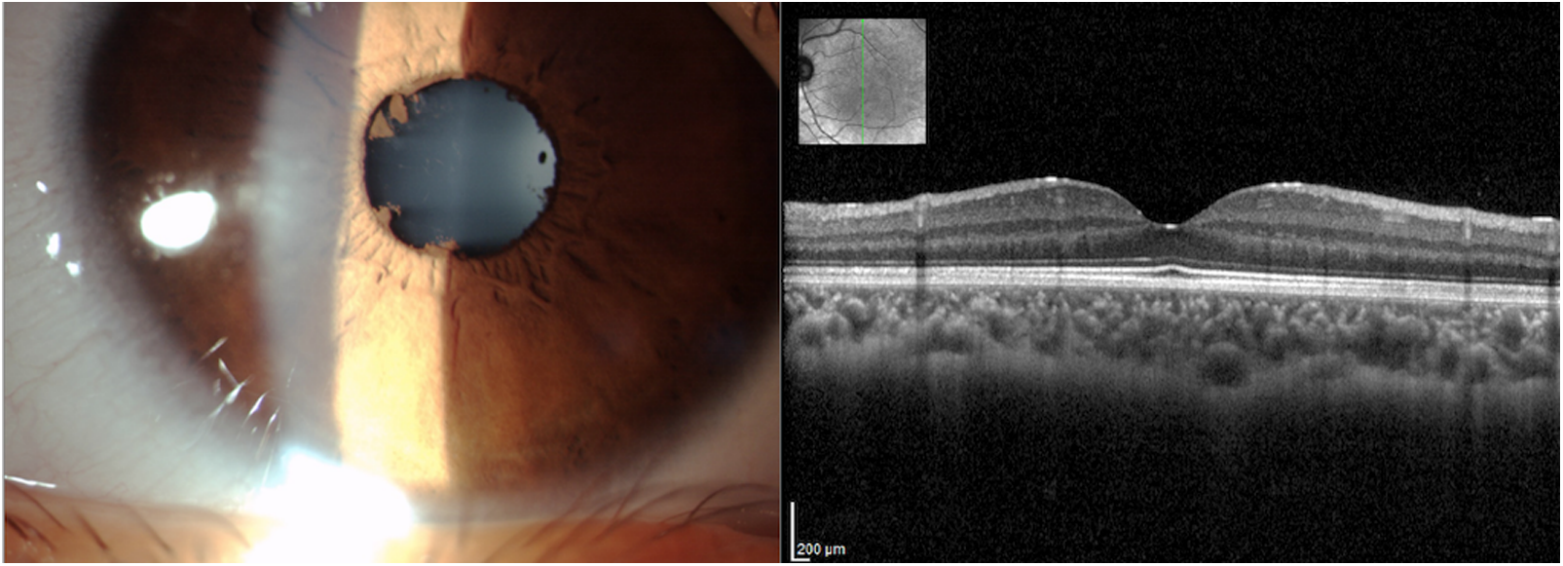
Multimodal imaging of the left eye of a 7-year-old boy with Prader–Willi syndrome (case 2). Anterior segment slit-lamp photography (left) showed no granulomatous deposits and posterior synechiae. Spectral-domain optical coherence tomography + infrared reflectance (right) displayed macular anatomy and thickness within normal limits.

**Figure 3:**
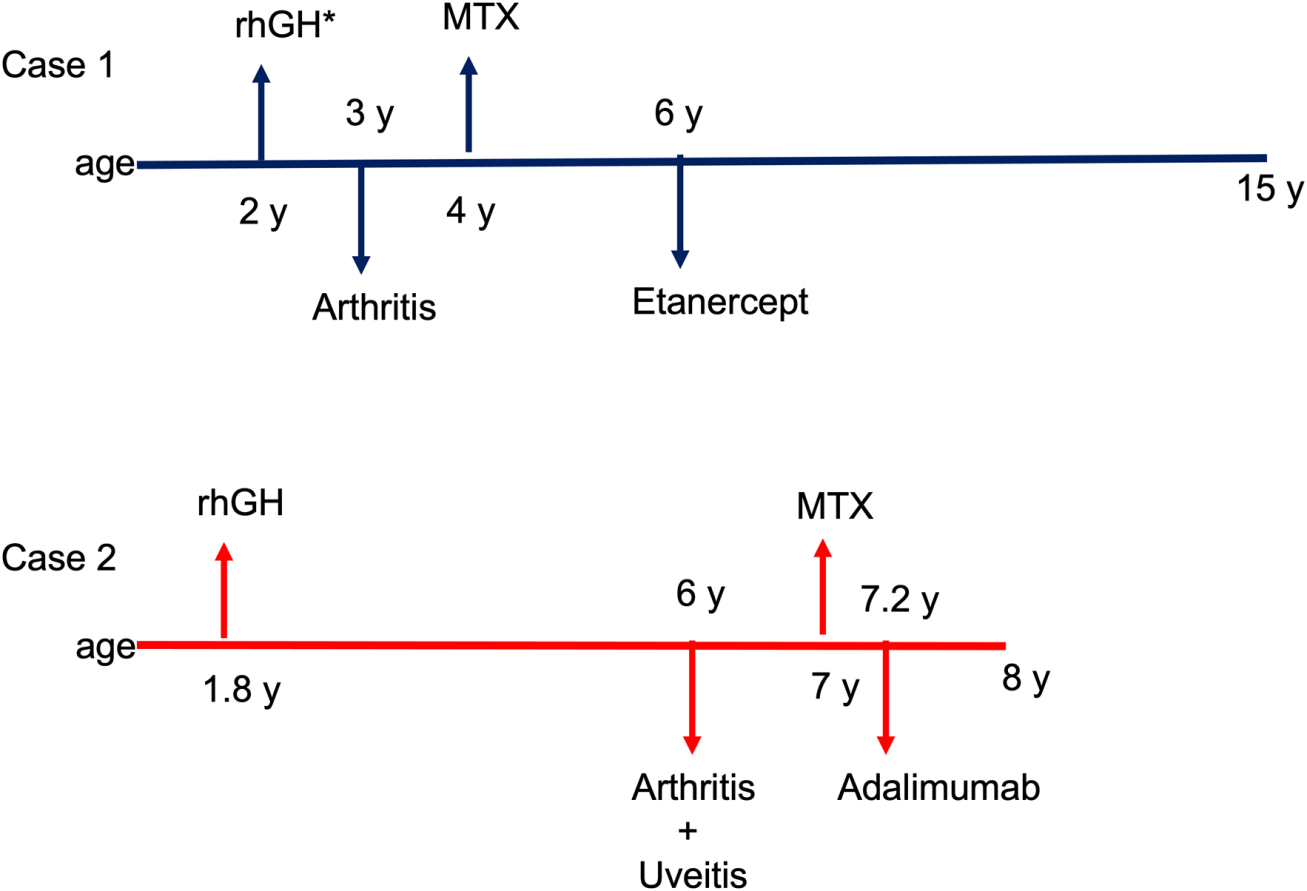
Timeline of clinical manifestations and treatments. RnGH, recombinant human growth hormone; MTX, methotrexate. *Treatment with rhGH was temporally discontinued for 15 months due to a diagnosis of ganglioneuroma.

At the age of 6 years, the patient was referred to our pediatric rheumatology outpatient clinic due to a 2-month history of painful swelling of the right knee, limited range of motion, and morning stiffness. The rheumatologic evaluation confirmed the presence of monoarthritis. Laboratory investigations showed mildly increased acute phase reactants, microcytic anemia, and ANA positivity at high titer (titer 1:1,280). Infectious and other causes of arthritis were excluded. Ultrasound confirmed the presence of joint effusion and synovial thickening (Figure 1), and synovial fluid analysis was consistent with inflammatory arthrosynovitis (white blood cell count of 14,200/µl) compatible with JIA. The patient underwent ophthalmological screening upon guidelines for patients with JIA. At the first eye evaluation, 1 month after the diagnosis of arthritis, a completed ophthalmological evaluation, including slit-lamp biomicroscopy, revealed the presence of inflammatory cells in the anterior chamber, posterior synechiae, band keratopathy, and initial cataract in both eyes (Figure 2), compatible with asymptomatic anterior bilateral uveitis. Intraocular pressure and fundus evaluation were normal. Subcutaneous MTX was begun, and topical corticosteroid therapy was instituted. Six months later, adalimumab was added due to persistent uveitis. At the last follow-up, 9 months later, the child experienced inactivity of arthritis and uveitis with normal growth (Figure 3).

## Discussion

To the best of our knowledge, chronic inflammatory arthritis and uveitis have not been described in patients with PWS. Our two patients showed early onset of arthritis lasting more than 6 weeks without a known underlying cause, positive ANA, raised inflammatory markers, leukocytes in synovial fluid analysis, joint effusion, and synovial thickening at ultrasound imaging (Table 1), complying with JIA diagnosis. Furthermore, ocular findings of asymptomatic anterior uveitis in case 2 are consistent with JIA-associated uveitis (6, 7). The clinical framework of the herein-reported patients fits the JIA classification criteria (8); therefore, the overall management of these patients (including treatment approach and uveitis screening schedule) followed the general recommendation for JIA. However, since JIA diagnosis is made after excluding other causes of arthritis, it might be argued that the concomitant presence of a predefined genetic syndrome could play a role in the development of joint inflammation. At present, there is no evidence of such speculation in PWS.

**Table 1:** Demographic and clinical features.

	Age at arthritis onset	Time to a pediatric rheumatology referral	Presence of morning stiffness	ANA	Elevates ESR and CRP	Pattern of arthritis	Uveitis	Treatment
Case 1	3 years	2 weeks	Yes	Positive^a^	CRP: 36.5 mg/L, ESR: 45 mm/h	Extended oligoarthritis	No	MTX + etanercept
Case 2	6 years	2 months	Yes	1:1,280	CRP: 14.6 mg/L, ESR: 46 mm/h	Monoarticular	Anterior, nongranulomatous, and complicated by posterior synechiae	MTX + adalimumab

^a^ ANA-IF: only semiquantitative assessment available.

The association with inflammatory arthritis has been previously investigated in Down syndrome (DS) (9). The DS-associated arthritis (DS-A) seems slightly different from JIA given the higher age at arthritis diagnosis (11 years), the higher rate of polyarticular-RF-negative arthritis, the frequent involvement of upper extremities, and the peculiar erosive course with a high burden of disability and joint damage (9).

Furthermore, patients with DS-A described in the series were ANA-negative, showed normal or low inflammatory markers, and none developed uveitis during the observation period. However, the diagnostic delay must be remarked as a potential bias. Indeed, more than half of a cohort of patients with DS-A (18 out of 33 patients; 55%) was identified through a musculoskeletal screening visit (out of 503 DS patients) (9).

Joint inflammation has also been reported in patients with Alagille syndrome (10). An international cohort reported 10 patients with Alagille syndrome and inflammatory arthritis. In this cohort, arthritis developed at 6.5 years of age and had a prevalent oligoarticular pattern (80%), with lower extremities more frequently affected. To note, two patients developed uveitis (10).

Inflammatory arthritis resembling JIA has also been described in Stickler syndrome (11). Various degrees of joint involvement have been described in metabolic diseases such as Gaucher disease and mucopolysaccharidoses; nevertheless, the articular disease in these disorders lacks inflammatory features (12, 13).

In a genetic syndrome setting, treating inflammatory arthritis might be challenging for several reasons, such as comorbidities, efficacy and safety of commonly used drugs, and finally low patient compliance (10, 14).

Jones et al. (14) reported higher rates of disease-modifying antirheumatic drug (DMARD) adverse events and ineffectiveness of biologic therapies in children with DS-A compared to canonical JIA patients (93% vs. 25% and 60% vs. 17%, respectively).

Ferrara et al. (10) reported a high rate of biological therapy use (80%) in patients with Alagille syndrome and inflammatory arthritis; this might be partially explained by the impossibility of using MTX, a DMARD of choice for inflammatory arthritis, in these children, given its potential hepatotoxicity .

In accordance with these data, both our patients required the combination of MTX and a TNF-a inhibitor to achieve disease remission.

Body mass index (BMI) is a well-known clinical parameter to influence JIA activity. Indeed, obesity may negatively impact disease course and treatment response in children with JIA (15, 16). On the other hand, JIA treatment (biologic and synthetic DMARDs) positively affects children's growth, fostering height gain with a stable BMI (17, 18). Focusing on PWS patients, these considerations are even more compelling.

Musculoskeletal manifestations are common in PWS and include ligamentous laxity, scoliosis, osteoporosis, limb misalignment, and foot abnormality (4). Muscle weakness and functional limitations of lower limb joints lead to a nonphysiological gait pattern in these children (3).

In this setting, intellectual disabilities, pain tolerance, and physical features such as obesity should be taken into consideration during standard evaluation. Indeed, food cravings and gain in body weight between the ages of 1 and 6 years play an important role in delayed motor development in most patients and might negatively impact the possibility of detecting signs of arthritis. The clinical examination might be challenging in PWS patients, and arthritis may be difficult to appreciate due to obesity and low patient compliance. In addition, gait abnormalities and musculoskeletal problems might be more easily attributed to noninflammatory manifestations of PWS, leading to diagnosis delay or misdiagnosis. Therefore, PWS children might benefit from periodic assessment of their musculoskeletal complaints to not miss possible inflammatory manifestations.

Although vision issues are a significant concern in children with PWS (19), chronic uveitis has never been described in these patients. Case 2 developed asymptomatic chronic anterior uveitis shortly after diagnosis of arthritis, compatible with uveitis, which is the typical extra-articular manifestation of JIA (20). Regular ophthalmological screening and early immunosuppressive treatment are mandatory to prevent vision-threatening complications and severe visual loss in patients with JIA-associated uveitis. A multidisciplinary approach between the pediatric rheumatologist and the uveitis specialist is of paramount importance in patients with JIA, particularly when there is a genetic-associated disorder.

In summary, we report the first clinical description of the association of PWS, chronic inflammatory arthritis, and anterior chronic bilateral uveitis compatible with JIA. We believe that it is crucial to raise awareness of this possible association among pediatricians since arthritis might be underestimated due to the high pain tolerance, behavior disturbances, and the presence of other musculoskeletal abnormalities in PWS patients. Furthermore, the possible development of asymptomatic uveitis makes the need for prompt diagnosis even more compelling.

## Data Availability

The raw data supporting the conclusions of this article will be made available by the authors without undue reservation.

## References

[1] Nicholls RD Saitoh S Horsthemke B. Imprinting in Prader–Willi and Angelman syndromes. Trends Genet. (1998) 14(5):194–200. 10.1016/S0168-9525(98)01432-2 9613204

[2] Gunay-Aygun M Schwartz S Heeger S O’Riordan MA Cassidy SB. The changing purpose of Prader–Willi syndrome clinical diagnostic criteria and proposed revised criteria. Pediatrics. (2001) 108(5):E92. 10.1542/peds.108.5.e92 11694676

[3] Cimolin V Galli M Vismara L Grugni G Camerota F Celletti C Gait pattern in two rare genetic conditions characterized by muscular hypotonia: Ehlers–Danlos and Prader–Willi syndrome. Res Dev Disabil. (2011) 32(5):1722–8. 10.1016/j.ridd.2011.02.028 21454046

[4] Shim JS Lee SH Seo SW Koo KH Jin DK. The musculoskeletal manifestations of Prader–Willi syndrome. J Pediatr Orthop. (2010) 30(4):390–5. 10.1097/BPO.0b013e3181da857d 20502241

[5] Butler MG Miller JL Forster JL. Prader–Willi syndrome—clinical genetics, diagnosis and treatment approaches: an update. Curr Pediatr Rev. (2019) 15(4):207–44. 10.2174/1573396315666190716120925 31333129 PMC7040524

[6] Heiligenhaus A Heinz C Edelsten C Kotaniemi K Minden K. Review for disease of the year: epidemiology of juvenile idiopathic arthritis and its associated uveitis: the probable risk factors. Ocul Immunol Inflamm. (2013) 21(3):180–91. 10.3109/09273948.2013.791701 23713827

[7] Paroli MP Abbouda A Restivo L Sapia A Abicca I Pivetti Pezzi P. Juvenile idiopathic arthritis-associated uveitis at an Italian tertiary referral center: clinical features and complications. Ocul Immunol Inflamm. (2015) 23(1):74–81. 10.3109/09273948.2013.855798 24329729

[8] Petty RE Southwood TR Manners P Baum J Glass DN Goldenberg J International League of Associations for Rheumatology classification of juvenile idiopathic arthritis: second revision, Edmonton, 2001. J Rheumatol. (2004) 31(2):390–2. 14760812

[9] Foley CM Deely DA MacDermott EJ Killeen OG. Arthropathy of Down syndrome: an under-diagnosed inflammatory joint disease that warrants a name change. RMD Open. (2019) 5(1):e000890. 10.1136/rmdopen-2018-000890 31245048 PMC6560675

[10] Ferrara G Giani T Lieberman SM Kremer C Hong S Indolfi G Alagille syndrome and chronic arthritis: an international case series. J Pediatr. (2020) 218:228–30.e1. 10.1016/j.jpeds.2019.10.042 31748120

[11] Dhaon P Das S Nolkha N. Arthritis in Stickler syndrome: inflammatory or degenerative? Int J Rheum Dis. (2017) 20(11):1785–7. 10.1111/1756-185X.12714 26178174

[12] Costi S Caporali RF Marino A. Mucopolysaccharidosis: what pediatric rheumatologists and orthopedics need to know. Diagnostics. (2022) 13(1):75. 10.3390/diagnostics13010075 36611367 PMC9818175

[13] Pastores GM Patel MJ Firooznia H. Bone and joint complications related to Gaucher disease. Curr Rheumatol Rep. (2000) 2(2):175–80. 10.1007/s11926-000-0059-x 11123056

[14] Jones JT Smith C Becker ML Lovell D, CARRA Registry Investigators. Down syndrome-associated arthritis cohort in the new childhood arthritis and rheumatology research alliance registry: clinical characteristics, treatment, and outcomes. Arthritis Care Res. (2021) 73(12):1739–45. 10.1002/acr.24418 33242376

[15] Giani T De Masi S Maccora I Tirelli F Simonini G Falconi M The influence of overweight and obesity on treatment response in juvenile idiopathic arthritis. Front Pharmacol. (2019) 10:637. 10.3389/fphar.2019.00637 31249526 PMC6582667

[16] Diaz-Cordovés Rego G Núñez-Cuadros E Mena-Vázquez N Aguado Henche S Galindo-Zavala R Manrique-Arija S Adiposity is related to inflammatory disease activity in juvenile idiopathic arthritis. J Clin Med. (2021) 10(17):3949. 10.3390/jcm10173949 34501396 PMC8432058

[17] Marino A Stagi S Simonini G Carli N Caparello MC Giani T Growth and body mass index in a cohort of patients with juvenile idiopathic arthritis: effects of second line treatments. Clin Exp Rheumatol. (2018) 36(5):929–33. 30148444

[18] Bharucha KN Brunner HI Calvo Penadés I Nikishina I Rubio-Pérez N Oliveira S Growth during tocilizumab therapy for polyarticular-course juvenile idiopathic arthritis: 2-year data from a phase III clinical trial. J Rheumatol. (2018) 45(8):1173–9. 10.3899/jrheum.170326 29961686

[19] Bohonowych JE Vrana-Diaz CJ Miller JL McCandless SE Strong TV. Incidence of strabismus, strabismus surgeries, and other vision conditions in Prader–Willi syndrome: data from the global Prader–Willi syndrome registry. BMC Ophthalmol. (2021) 21(1):296. 10.1186/s12886-021-02057-4 34380467 PMC8359621

[20] Sen ES Ramanan AV. Juvenile idiopathic arthritis-associated uveitis. Clin Immunol. (2020) 211:108322. 10.1016/j.clim.2019.108322 31830532

